# The effect of human menstrual blood-derived stem cells on ovarian folliculogenesis, angiogenesis and collagen volume in female rats affected by the polycystic ovary syndrome

**DOI:** 10.1186/s13048-023-01244-9

**Published:** 2023-08-22

**Authors:** Ali Sarhadi Roodbari, Somayeh Solhjoo, Maria Grazia Palmerini, Mahna Mansouri, Massood Ezzatabadipour

**Affiliations:** 1 Anatomical Sciences Department, Afzalipour School of Medicine, Kerman University of Medical Sciences, Kerman, Iran; 2 Department of Life, Health and Environmental Sciences, University of L’Aquila, L’Aquila, Italy

**Keywords:** Polycystic ovary syndrome, Menstrual blood stem cells, Folliculogenesis, Angiogenesis, Fibrosis

## Abstract

**Background:**

Infertility is one of the common problems among couples, affecting millions of people worldwide. Polycystic ovary syndrome (PCOS) is one of the main causes of infertility in women and is associated with abnormal folliculogenesis, angiogenesis and fibrosis. Common treatments may lead to numerous adverse effects on the patient’s quality of life. The present study aimed to investigate the effects of human menstrual blood-derived stem cells on the ovarian histology of a PCOS model of Wistar rats.

**Results:**

Based on the Papanicolaou test and H&E staining results, the number of primary, secondary and antral follicles in the PCOS and PCOS-Sham groups significantly increased compared to the control group, while they significantly decreased in the PCOS + Stem cells group compared to the PCOS and PCOS-Sham groups. Further, the number of atretic follicles in both PCOS and PCOS-Sham groups significantly increased in comparison with the control group and decreased in the PCOS + Stem cells group, compared to the two mentioned groups. Moreover, the Graafian follicles number was decreased in the PCOS and PCOS-Sham groups to significantly increase in the PCOS + Stem cells group. Based on Masson’s trichrome staining, the number of blood vessels in PCOS and PCOS-Sham groups significantly increased compared to the control group, while a decrease was observed in the PCOS + Stem cells group, compared to PCOS and PCOS-Sham groups.

**Conclusion:**

The administration of MenSCs improved folliculogenesis in rats with polycystic ovaries. Also, MenSCs could ameliorate PCOS symptoms by improving fibrosis as well as angiogenesis and weight gain.

## Introduction

Infertility, known as one of the most common problems among couples, is affecting millions of people worldwide [1]. Different factors cause infertility in women and, among them, polycystic ovary syndrome (PCOS) is considered one of the main factors [2]. PCOS is one of the most common endocrine-related metabolic disorders in women with high-level androgens, anovulation, and polycystic ovaries. It affects 5–20% of women of reproductive age [3], with complications including menstrual irregularities, infertility, hirsutism and cardiovascular diseases (CVD) [4].

This syndrome is a complex disorder with unknown pathophysiology [5] influenced by genetic, environmental, and immunological factors in its development [6]. Evidence indicates the relationship between the chronic inflammatory condition in PCOS patients and its pathogenesis. In this regard, it has been demonstrated that the expression of some acute and inflammatory phase proteins, such as interleukin 1 (IL-1), interleukin 6 (IL-6), tumor necrosis factor α (TNF-α), and serum C-reactive protein (CRP) are increased in PCOS patients, with a detrimental effect on ovarian function, ovulation, fertilization and implantation [7]. For these reasons also, PCOS has been referred to as a low-grade chronic inflammatory disorder, intensified by obesity [8, 9].

Women with PCOS have a large number of small antral follicles (more than twice that in normal ovaries) at the periphery of the ovary but many do not proceed in the physiological growth leading to ovulation disorders or anovulation [5]. Several reports indicated the increase in fibrotic tissue and the accumulation of type I collagen in the ovarian stroma and type IV collagen in basement membranes in the ovaries of PCOS women and mice. All are symptoms of polycystic ovary and impaired reproductive function [10–13]. Moreover, it is well known that angiogenesis plays an essential role in proper follicle growth and ovulation [14] and alterations of ovarian angiogenesis occur in various conditions such as PCOS, uterine bleeding, infertility, and endometriosis [15–17].

Some undesirable symptoms of PCOS can be ameliorated by decreasing blood testosterone levels (glucocorticoids), regulating the menstrual cycle (oral contraceptives), and inducing ovulation (clomiphene citrate). Nonetheless, these treatments may lead to serious side effects affecting the patient’s quality of life. Despite advances in existing therapies, regenerative medicine is representing a valid alternative for treatments. Tissue engineering and stem cells have been used in this field for various disorders in the uterus, vagina and ovaries [18].

Stem cell therapy is known as a promising strategy to treat damaged tissues or restore their function [19]. Stem cells - including embryonic, mesenchymal and induced pluripotent stem cells (IPSC) – may be applied to regenerate damaged tissues [20]. Human mesenchymal stem cells (hMSCs) have low immunogenicity and tumorigenesis. Therefore, the use of these cells and the induction of xenogeneic animals have become a new method to evaluate the efficacy of hMSCs, thus providing preclinical data for the design of clinical trials in PCOS patients [21, 22].

Mesenchymal stem cells (MSCs) have immunomodulatory properties that can inhibit T cell proliferation and reduce T cell cytotoxicity. Additionally, they prevent B cell proliferation and maturation and reduce the production of antibodies (IgG, IgM). Because of these properties, MSCs are promising strategies to improve treat-resistant autoimmune diseases such as rheumatoid arthritis (RA), systemic lupus erythematosus (SLE), atopic dermatitis (AD), systemic sclerosis, and type I diabetes [6].

MSCs can be extracted from various adult tissues, such as blood, bone marrow and adipose tissue. Another source of stem cells is represented by those derived from menstrual blood (MenSCs), introduced by Meng and colleagues in 2007. These cells were originally known as endometrial regenerating cells and, due to advantages such as easy collection, high proliferation rate, pluripotency, and lower immune response, were successfully applied [19]. These cells can be differentiated into different lineages in vitro, including osteocytes, chondrocytes, adipocytes, endothelial cells and oocyte-like cells [23, 24]. Menstrual blood-derived stem cells exert their therapeutic effects mainly by interacting with various immune cells, effectively secreting numerous cytokines, and also targeting damaged areas [24]. Considering the evidence of human menstrual blood-derived stem cell transplantation in animal models of xenograft transplantation and the positive effects of mesenchymal stem cells in improving ovarian function in animal models and the few studies that have been conducted in this field, this study aims to investigate the paracrine effect of stem cells derived from menstrual blood on folliculogenesis, angiogenesis and ovarian collagen volume in a PCOS model of female Wistar rats as.

## Materials and methods

### Experimental animals

In the present study, 40 female Wistar rats (weighing approximately 230−200 g) were purchased from the Royan Research Institute in Tehran. All procedures were conducted under the approval of the ethics committee of the Kerman Medical School (IR.KMU.AH.REC.1399.132). The animals were kept at 21–23 ºC and 12/12 light-dark cycle with free access to standard diet and water.

### Estrous cycle assessment

After performing the Papanicolaou test, specific staining and light microscopy assessment were performed to evaluate the estrous cycle synchrony.

### Experimental groups

To induce PCOS in female rats, 1mg/kg letrozole (Letrax, Abu Reihan Pharmacy, Iran) dissolved in 1% carboxymethylcellulose (CMC), was orally administrated for 21 consecutive days [25]. Animals were then divided into five groups (*n* = 8) including, Control (without intervention); Sham (receiving the CMC, orally); PCOS (received letrozole with a dose of 1 mg/kg); PCOS + Stem cells (ovaries of PCOS-induced animals were injected with 2 × 10^6^ MenSCs); and PCOS-Sham group (a needle was inserted into the ovary without any administration).

### Extraction and culture of human MenSCs

First, 2–3 ml of menstrual blood samples were collected from the second day of menstruation from five healthy women aged 20–30 with regular menstrual cycles, without a history of sexually transmitted diseases and diseases such as PCOS, premature ovarian failure) POF(, premature ovarian insufficiency) POI(. This procedure was conducted according to the informed consent approved by the ethics committee of Kerman University of Medical Sciences (Kerman, Iran). MenSCs were isolated from menstrual blood samples collected from menstrual cups (lunette, Finland). Then, Samples were washed with PBS, and MenSCs isolation was performed with Ficoll solution based on gradient concentration. Cells in the middle layer after centrifugation (30 min and 1800 RPM) were collected by using a Pasteur pipette. The obtained cells were transferred into a flask containing DMEM/F 12 (10% FBS, 100 mg streptomycin, and 100 U /ml penicillin) and incubated (37° C with 5% CO_2_) to obtain an 80–90% confluency [26].

### Flow cytometry

The cells in passage 3 were suspended in phosphate-buffered saline (PBS) and stained with the antiCD45, CD34, CD105, and CD90 antibodies, for 30 min at 4 °C (all from BD-Biosciences, USA) to characterize the linage of isolated cells. To compare the results of the different tests, a similar isotype-matched control antibody was applied. For each group, 10,000 incubated cells were analyzed with FACSCalibur flow cytometer and Cellquest Pro software (version 5.1).

### PKH26 labeling of human MenSCs

Before transplantation, human MenSCs were labeled with PKH26 fluorescent dye (Sigma Aldrich, USA) according to the manufacturer’s protocol. Briefly, after detachment, MenSCs (2 × 10^6^), were washed in a serum-free medium and suspended in 1 ml of PBS. Four microliters of PKH26 solution were diluted in PBS (1:1) and the cells were immediately incubated with the prepared solution for 25 min at 25 °C. Then, the labeling process was completed by adding 2 ml of fetal bovine serum (FBS). Finally, the cells were washed with an enriched culture medium (Denazist, Iran) [6].

### Human MenSCs transplant

Following PCOS induction in the PCOS + Stem cells group, animals were anesthetized with ketamine and xylazine as was early described [27]. Briefly, after shaving the supra-flank area and finding the twelfth rib, these regions were completely prepped, as well as the incision area was disinfected with povidone-iodine. First, the ovaries were carefully exposed and human MenSCs (1 × 10^6^ cells were suspended in 40 µl of PBS) were injected into the ovarian tissue with a 33-gage needle. Then, the abdominal wall was sutured and the surgical site was disinfected by using tetracycline ointment. Finally, two weeks after the MenSCs transplant, animals were sacrified and ovaries were removed for histological evaluations.

### Ovarian and body weight measurement

The body weight of animals was measured before and after the treatment. In addition, following the anesthesia of animals in five groups [by i.p. injection of ketamine (40 mg/kg) + xylazine (5 mg/kg)], both ovaries were removed and their weights were recorded. Then, the ovaries were fixed in a 10% formalin solution for further evaluation.

### Assessment of the number of follicles and ovarian morphometry by H&E staining

Fixed samples were dehydrated using an ascending series of ethanol. Then, samples were clarified with xylene and immersed in paraffin. Paraffin blocks were cut (5 μm) under a microtome (Miro DS 4055, Iran) and sections were stained with hematoxylin and eosin (H&E) to assess the number of ovarian follicles and corpora lutea. To these aims, a Sect. (5 μm) was selected per ten cross sections. The sections were selected at a distance of 50–60 μm [28]. Then, the number of follicles (primordial, primary, secondary, antral, Graafian, atretic), cysts and corpora lutea were counted [29]. Cysts were defined as by a large antrum with attenuated granulosa cell layer, also flattened granulosa cells that faced the antrum. Theca interna becomes hypertrophic [30].

### Counting the number of blood vessels

Masson’s trichrome staining [31] was used to count blood vessels in different groups [32]. To achieve these goals, sections were chosen between 5 and 20 μm in the next section, then, the number of blood vessels was counted.

### Assessment of the fibrosis by stereological studies

The ovaries were dehydrated and clarified with an automated tissue processor and immersed in paraffin. After blocking, the section series method was applied to prepare uniform random sections. Briefly, paraffin blocks were cut in sections with 5 and 20 μm thicknesses. The sections were stained by using Masson’s trichrome staining method. Moreover, sections from each animal were randomly selected for histological assessment. Eventually, the total volume of the ovary, the collagen and vessels volume as well as the area of blood vessels were calculated with Cavalieri’s principle [33].

Pictures were randomly taken from the 5 μm thickness sections of the ovaries and then the points (The technique of counting points according to Cavalieri’s principle) were randomly placed on the images by using a light microscope (Nikon Eclipse 50i) and Image j software 1.44 version. Then the points were counted and the total volume of the ovary was estimated using the following formula:

$$V Total=\frac{{\sum }_{i=0}^{n}P\times a\left(P\right)\times t}{{M}^{2}}$$

P is the total number of points counted in the selected sections. A (P) is the area associated with the positive point, t is the thickness of the selected sections and M is the magnification of the light microscope.

After measuring the total volume of the ovary, the reference volume of collagen and blood vessels was also measured. In this way, the images of the ovary were taken with a magnification of 20X and from 4 fields, and the points were randomly placed on the photos using Image J software. Then the points intersected with collagen tissue and blood vessels were counted and the volume of collagen and blood vessels was obtained from the following formula:

$$Vv=\frac{\frac{{\sum }_{i=0}^{n}P\times a\left(P\right)\times t}{{M}^{2}}}{V Total}$$

### Statistical analysis

The data were analyzed using Graph pad Prism 8 software. The one-sample Kolmogorov-Smirnov test was used to check the normality of the data distribution, then the property data of the normal distribution were analyzed with One-way ANOVA and Post Hoc Tukey test and the non-normally distributed data with the Non-parametric Kruskal-Wallis test. P < 0.05 were considered to determine the significance level of difference between groups.

## Results

### Estrous cycle assessment and PCOS model confirmation

**Fig. 1:**
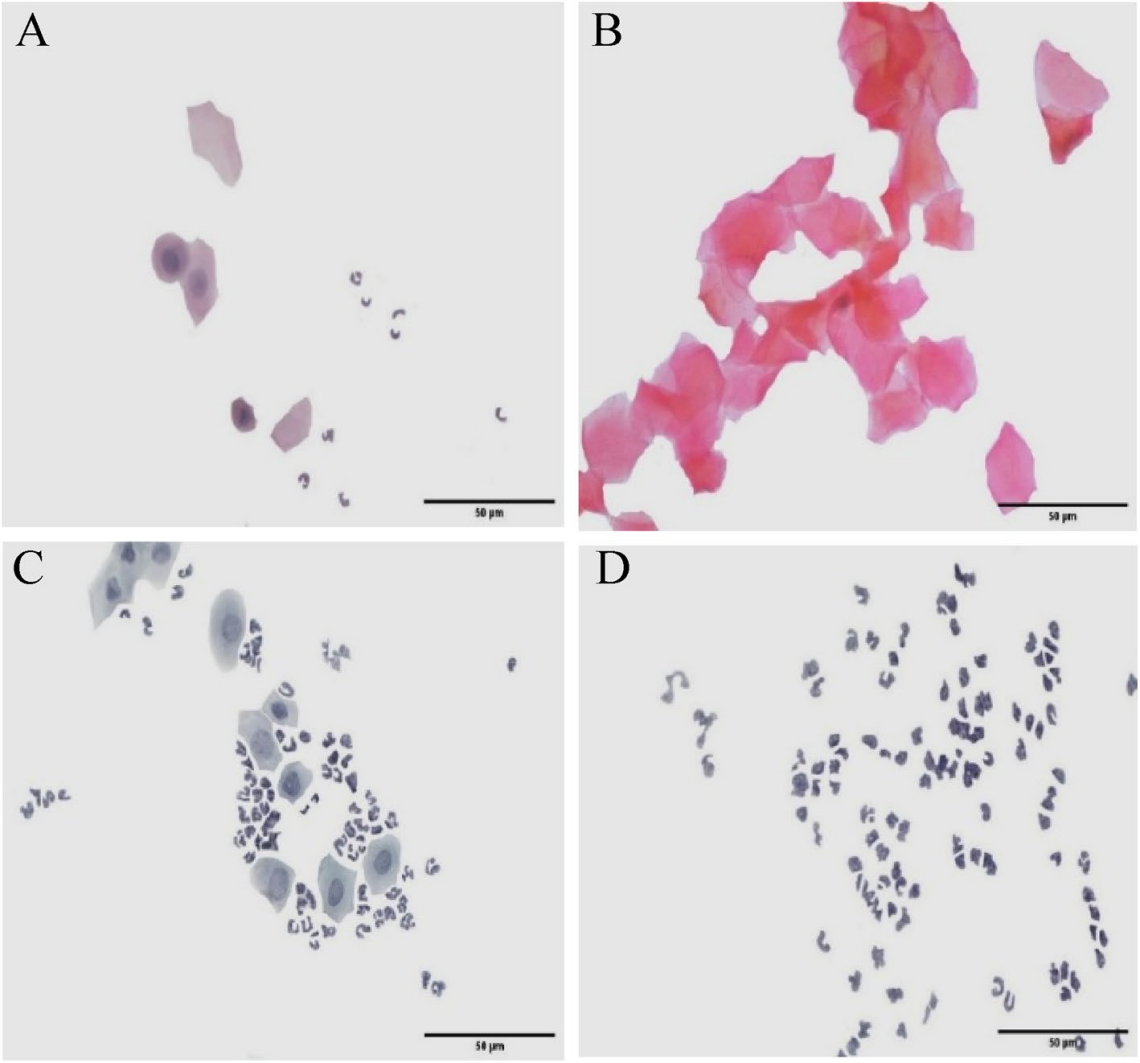
Vaginal cytology of animals in the control group. **A**) nucleated epithelial cells, cornified epithelial cells and leukocytes at the proestrus phase; **B**) cornified epithelial cells at the estrous phase; **C**) nucleated epithelial cells and leukocytes at the metestrus phase; **D**) numerous leukocytes at the diestrus phase. **E**) continuated diestrus phase in PCOS grup. Papanicolaou staining, scale bar: 50 μm

All female rats in the control group evidenced a normal estrus cycle (characterized by proestrus, estrus, metestrus and diestrus), by using vaginal cytology and Pap smear (Fig. 1). Differently, the vaginal smear cytology of letrozole-treated rats displayed an irregular estrous cycle with a continuation of the diestrus phase. Therefore, these data evidenced that the PCOS model was successfully induced.

### Cell culture and MenSC characterization

**Fig. 2:**
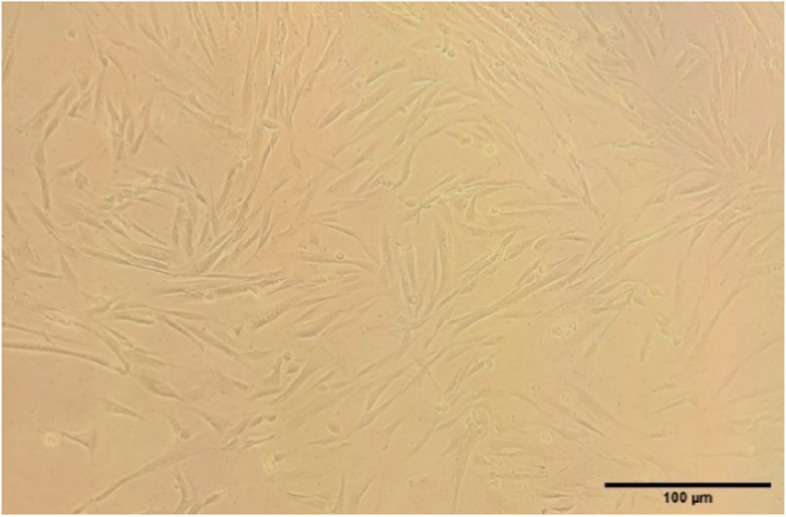
Morphology of human menstrual blood stem cells. Scale bar = 100

Cultured MenSCs had a spindle-shaped appearance, They were observed sticking to the bottom of the dish (Fig. 2), which had a high proliferation ability and reached in about two weeks to confluency of 80%.

### Confirmation of mesenchymal stem-cell markers

**Fig. 3:**
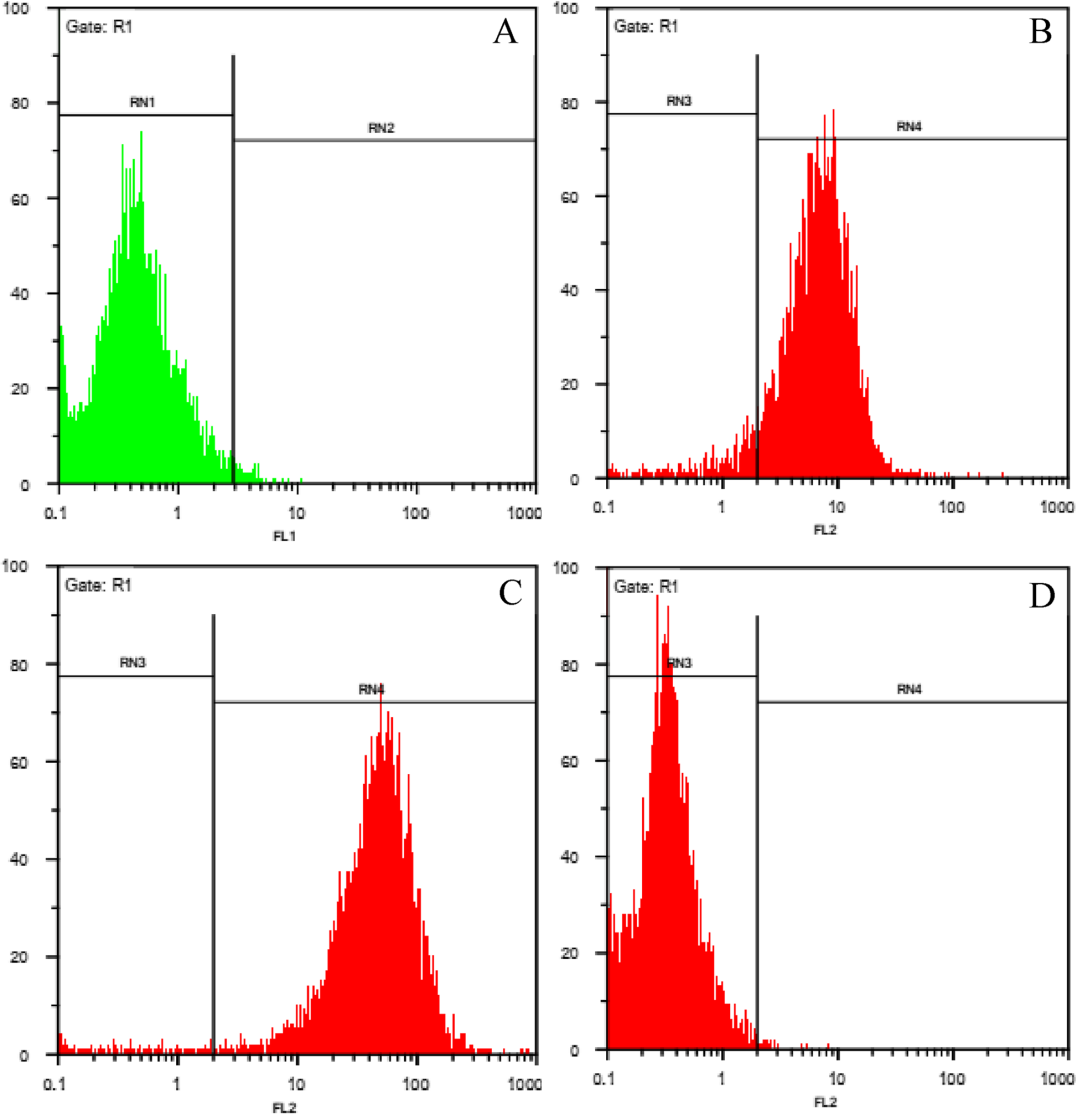
The expression of surface arkers in MenSCs. **A**) CD45 **B**) CD105 **C**) CD90 **D**) CD34. RN: are regions around populations of cells with common characteristics

The profile of cell surface antigens in stem cells derived from menstrual blood in passage 3 was evaluated. All human MenSCs significantly expressed CD90 (97.60%) and CD105 (93.33%) markers (Fig. 3A-D), while they were negative for CD34 (99.70%) and CD45 (99.47%).

**Fig. 4:**
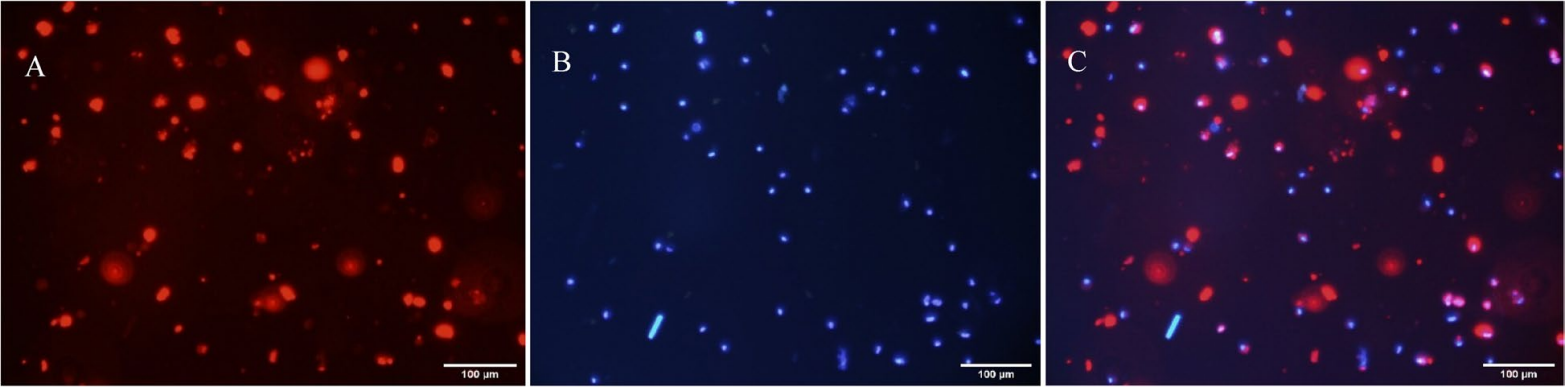
Florescence micrograph of PKH26 and Hoechst labelled MenSCs. **A**) PKH26 stained the cell membranes (red points) **B**) Hoechst stained the cell nuclei (blue points) C) Merged. CM (confocal microscopy). Scale bar = 100 um

**Fig. 5:**
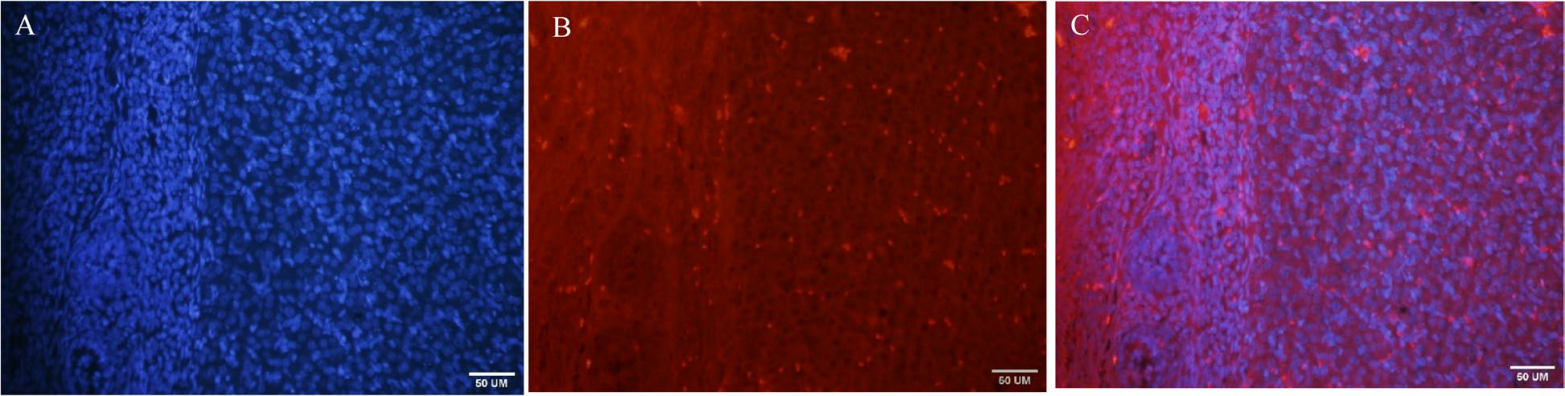
Homing of KH26 labelled Menscs in PCOS ovary. **A**) Nuclei were labelled with Hoechst; **B**) Cell membranes labelled withPKH26; **C**) Merged. CM, scale bar = 50 um

Labeling of the MenSCs wih PKH26 Homing of MenSCs cells after intraovarian injection was evaluated using fluorescent PKH26 staining and Hoechst. Then the cells were examined under a Fluorescence microscope (Olympus IX71, Japan) for injection into the ovaries of the animal (Figs. 4 and 5).

### Angiogenesis assessment

**Fig. 6:**
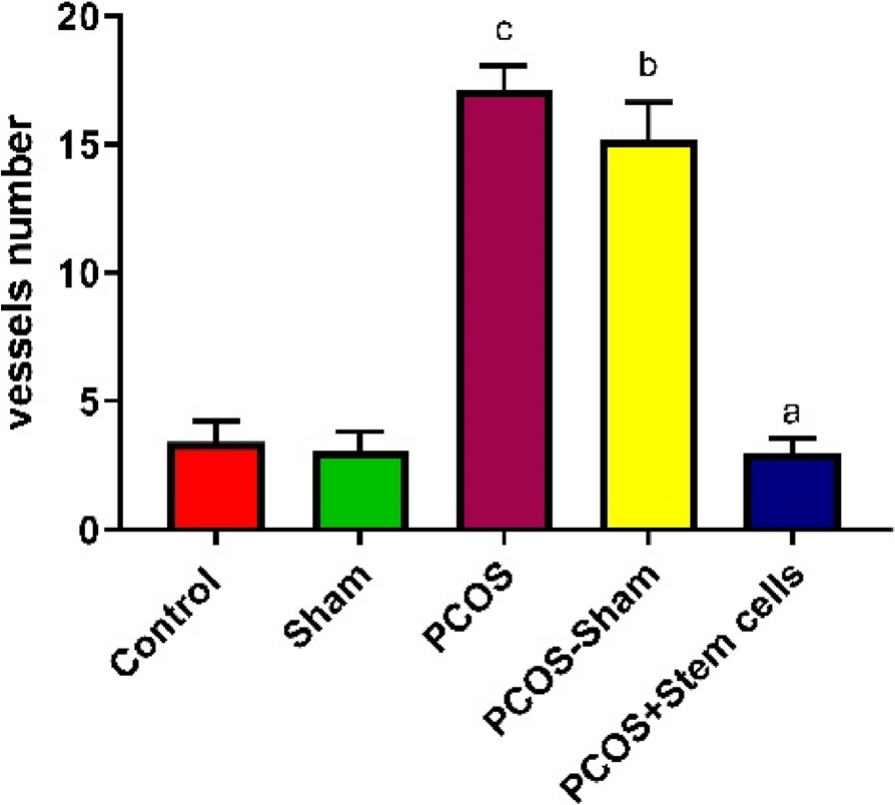
The number of blood vessels in the experimental groups with counting points based on the Cavalieri`s principle (**a**: vs PCOS-sham and PCOS; **b**: vs Sham + Control; **c**: P vs Sham + Control)

**Fig. 7:**
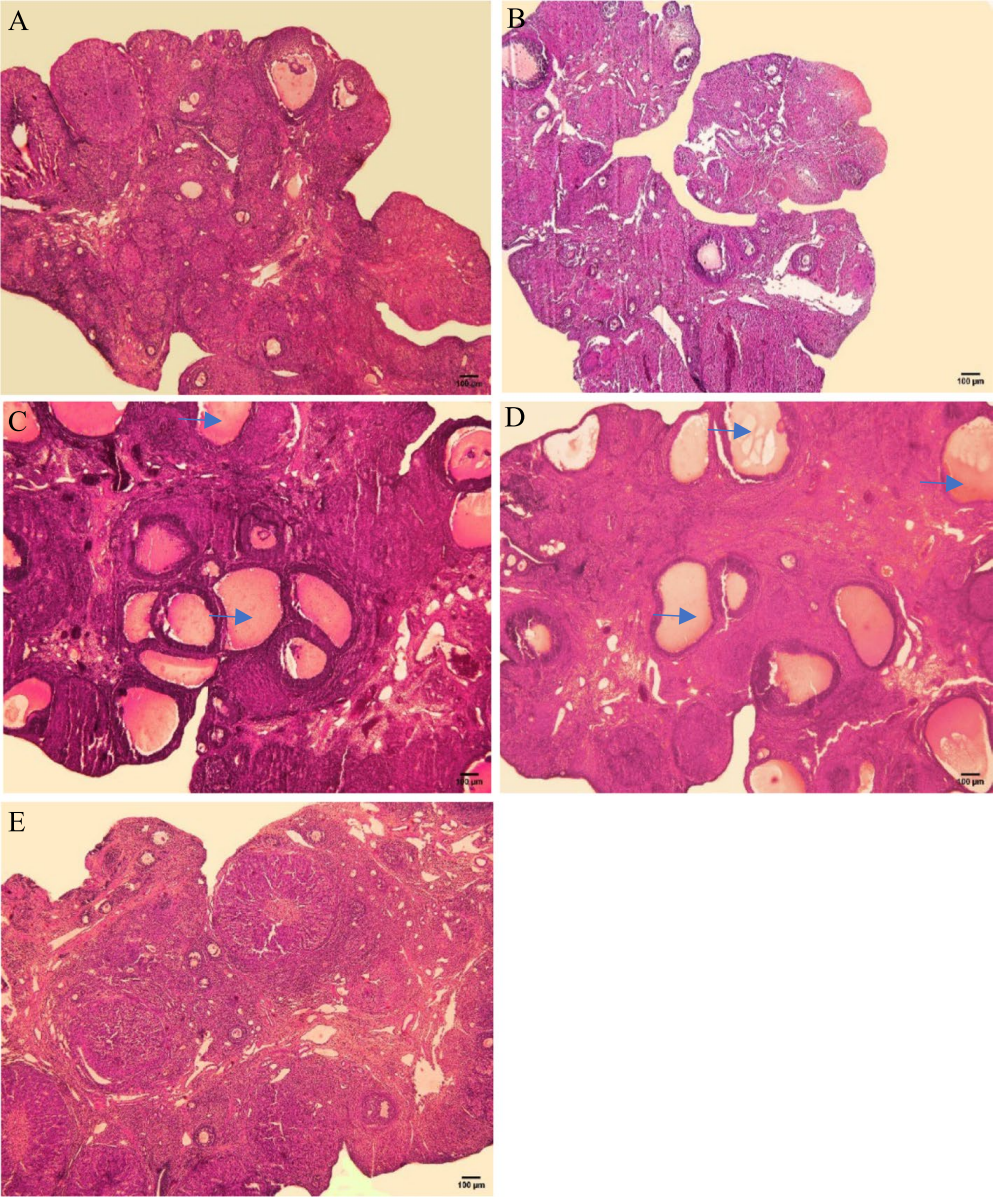
Histopathological changes in the ovaries of the control and experimental groups. **A**) control group; **B**) sham group (CMC); **C**) PCOS group; **D**) PCOS-Sham group; **E**) PCOS+Stem cells group. The control group showed normal ovarian morphology, while the PCOS and PCOS-Sham groups showed the presence of many cysts (blue arrows). In the PCOS+Stem cells group, the ovarian structure seemed more similar to controls, with an increase in the number of follicles and a decrease in cysts. H&E staining. LM, scale bar:100 um

The number of blood vessels in the PCOS (17.10 *P* ≤ 0.0001) and PCOS-Sham (15.17, *P* ≤ 0.0001) groups significantly increased compared to the Control group (3.40). On the contrary, the number of blood vessels in the PCOS + Stem cells group (2.97) decreased significantly respect to PCOS (*P* ≤ 0.0001) and PCOS-Sham (*P* ≤ 0.0001) (Figs. 6 and 7).

### H&E staining and evaluation of folliculogenesis

Six fields in each section were evaluated to count follicles in different groups. Ovaries from Control and Sham groups showed a normal morphology, with primordial, primary, secondary, Graafian, some antral follicles and visible corpora lutea in the cortex (Fig. 7A,B), while the PCOS and PCOS-Sham groups showed many cysts (Fig. 7C, D). Differently, in the PCOS + Stem cells group the ovarian structure seemed to be ameliorated, with an increased number of follicles, and a decreased number of cysts (Fig. 7E).

**Fig. 8:**
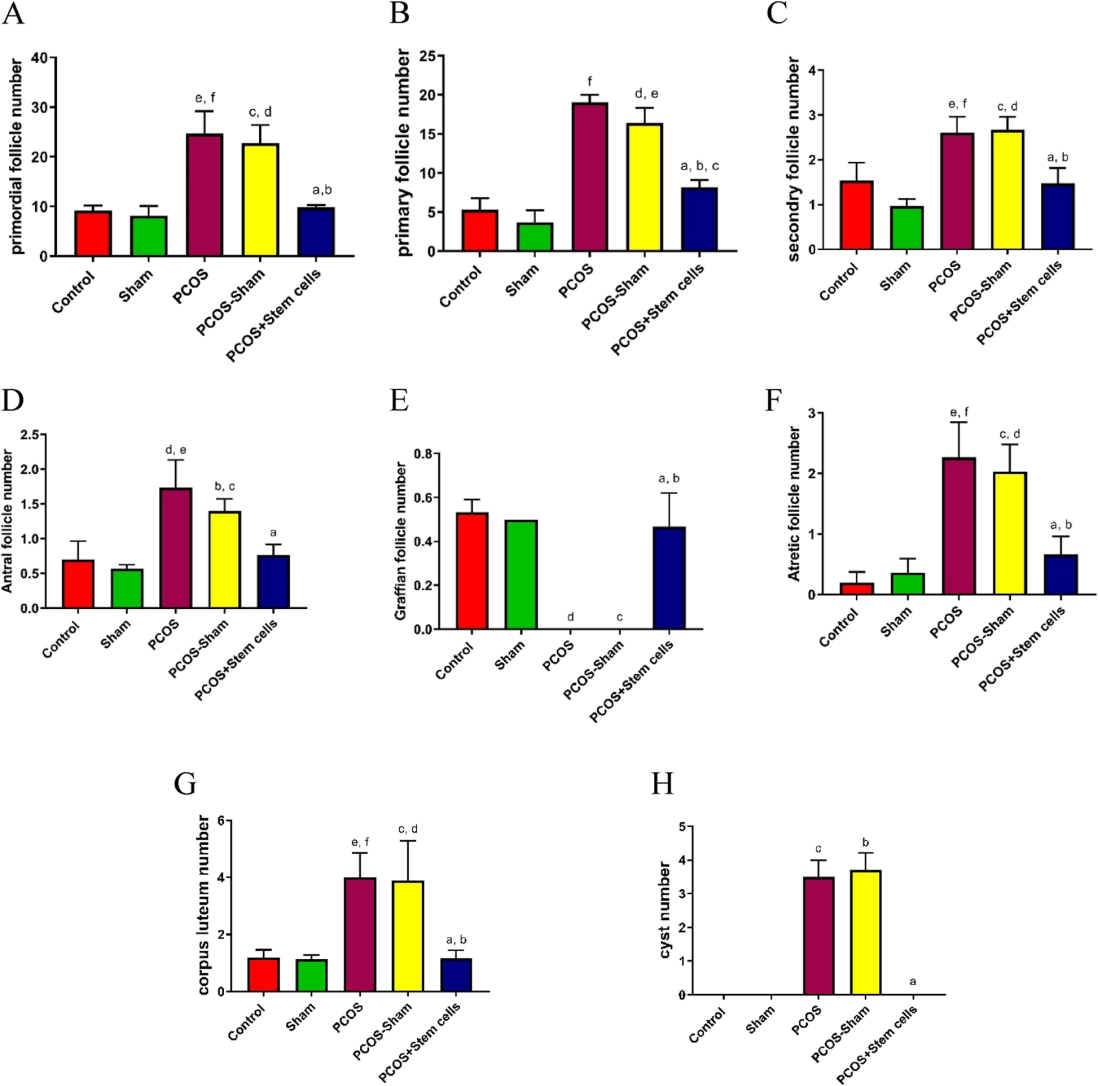
Folliculogenesis in experimental groups. **A**) Primordial follicles number. **B**) Primary follicles number. a: vs PCOS; b: vs PCOS-Sham; c: vs Sham; d: vs Control; e: vs Sham; f: vs Control and Sham; **C**) Secondary follicles number. a: vs PCOS; b: vs PCOS-Sham; c and e: vs Control; d and f: vs Sham. **D**) Antral follicles number. a: vs PCOS; b and d: vs Control; c and e: vs Sham. **E**) Graafian follicles number. a: vs PCOS; b: vs PCOS-Sham; c and d: vs Control and Sham. **F**) Atretic follicles number. a: vs PCOS; b: PCOS-Sham; c and e: vs Control; d and f: vs Sham. **G**) Corpus luteum number.  a = *P* ≤ 0.05 vs Sham; b = *P* ≤ 0.05 Sham; c = *P* ≤ 0.05 vs Control; d = *P* ≤ 0.05 vs Control. **H**) Cyst. a: vs PCOS and PCOS-Sham; b and c: vs Control and Sham

The number of primordial follicles in the ovaries of PCOS and PCOS-Sham groups, There was no significant difference compared to other groups (Fig. 8A).

The number of primary follicles in the ovaries of PCOS (19.03) and PCOS-Sham groups (16.40) were significantly higher than those in the control (5.30, vs. PCOS and PCOS-Sham; *P* ≤ 0.0001) and sham groups (3.70, vs. PCOS and PCOS-Sham; *P* ≤ 0.0001). Nonetheless, the number of primordial follicles in the PCOS+Stem cells group (8.16) significantly decreased compared to PCOS (*P* ≤ 0.0001) and PCOS-Sham (*P* ≤ 0.001) groups, and increased respect to the sham group (*P* ≤ 0.05) (Fig. 8B).

The number of secondary follicles in the ovaries of PCOS (2.6) and PCOS-Sham groups (2.66) were significantly higher than those in the Control (1.53, vs. PCOS and PCOS-Sham; *P* ≤ 0.05) and sham groups (0.96, vs. PCOS and PCOS-Sham; *P* ≤ 0.001). Differently, the number of secondary follicles in the PCOS + Stem cells group (1.46) significantly decreased compared to PCOS (*P* ≤ 0.05) and PCOS-Sham (*P* ≤ 0.01) groups (Fig. 8C).

As shown in Fig. 8D, the number of antral follicles in PCOS (2.6) and PCOS Sham (2.66) ovaries were significantly higher than that in the control (1.53, vs. PCOS: *P* ≤ 0.01 and PCOS-Sham; *P* ≤ 0.05) and sham groups (0.96, vs. PCOS: *P* ≤ 0.01 and PCOS-Sham; *P* ≤ 0.05). On the contrary, the number of antral follicles in the PCOS+Stem cells group (1.46) significantly decreased compared to the PCOS group (*P* ≤ 0.01).

No Graafian follicles were found in the PCOS (0) and PCOS-Sham (0) groups compared to the control (0.53, *P* ≤ 0.0001) and Sham (0.5, *P* ≤ 0.0001) groups. The number of Graafian follicles significantly increased in the PCOS + Stem cells group compared to PCOS (*P* ≤ 0.001) and PCOS-Sham (*P* ≤ 0.001) groups (Fig. 8E).

The number of atretic follicles in the PCOS (2.26) and PCOS-Sham (2.03) groups significantly increased compared to the control (0.2, *P* ≤ 0.001) and Sham (0.36, vs. PCOS: *P* ≤ 0.001 and vs. PCOS-Sham: *P* ≤ 0.01) groups. Additionally, the reduction in the number of atretic follicles in the PCOS + Stem cells group (0.67, *P* ≤ 0.01) was significant compared to the PCOS and PCOS-Sham groups (Fig. 8f).

The number of corpora lutea in the PCOS (1.1) and PCOS-Sham (0.9) groups significantly decreased compared to the Control (4.8, *P* ≤ 0.05) and Sham (4.3, *P* ≤ 0.05) groups (Fig. 8G).

Finally, the number of cysts in the PCOS (3.5) and PCOS-Sham (3.7) groups significantly increased compared to the Control (0, *P* ≤ 0.0001) and Sham (0, *P* ≤ 0.0001) groups; the reduction in the PCOS + Stem cells group (0, *P* ≤ 0.0001) was significant compared to the PCOS and PCOS-Sham groups (Fig. 8H).

### Measurement of the volume and area of collagen and blood vessels

**Fig. 9:**
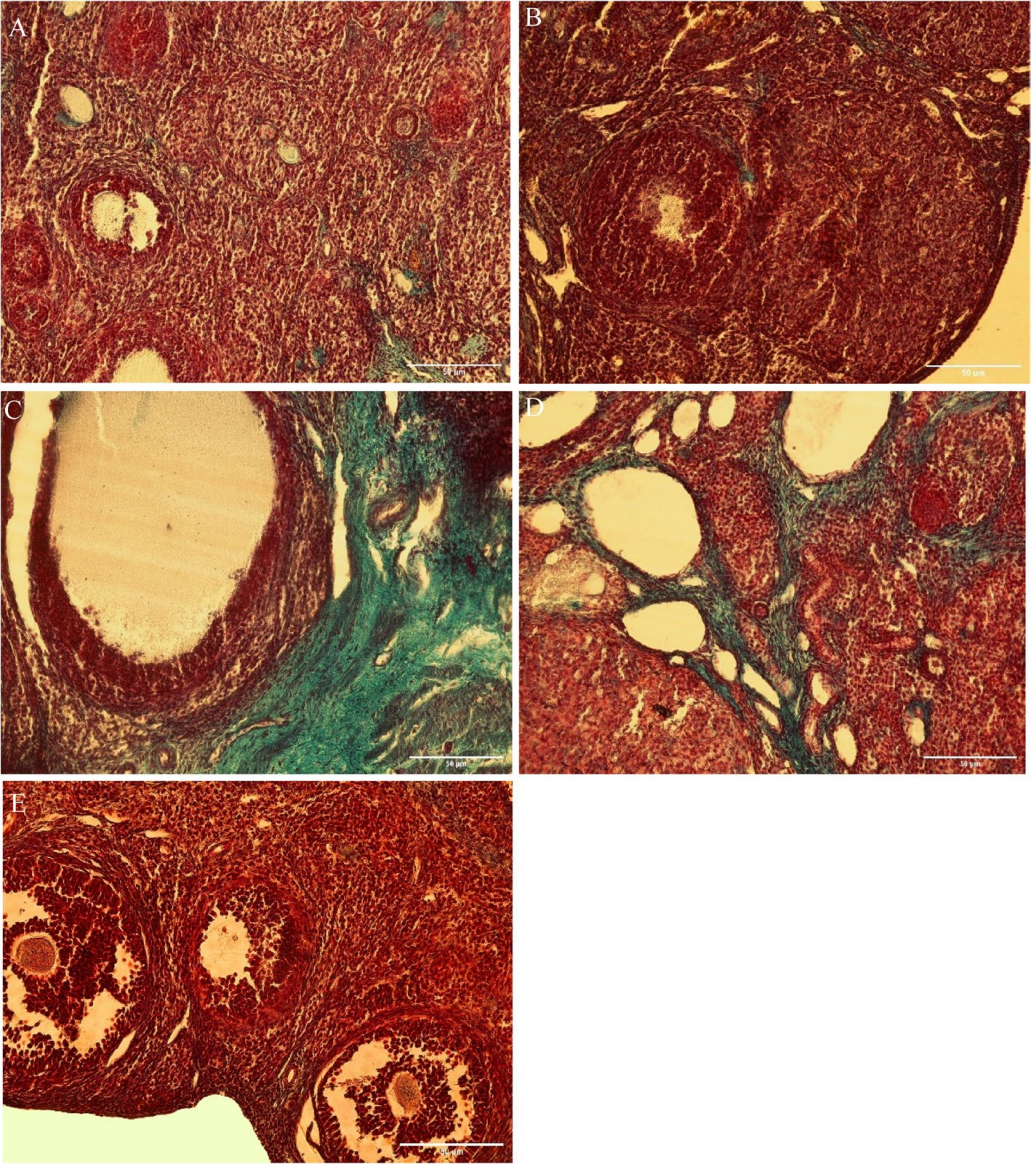
Histopathological changes in the ovaries of the control and experimental groups (Masson trichrome staining, scale bar, 50 µm. **A**) Control group; **B**) Sham group (CMC); **C**) PCOS group; **D**) PCOS Sham group; **E**) PCOS + Stem Cells group

**Fig. 10:**
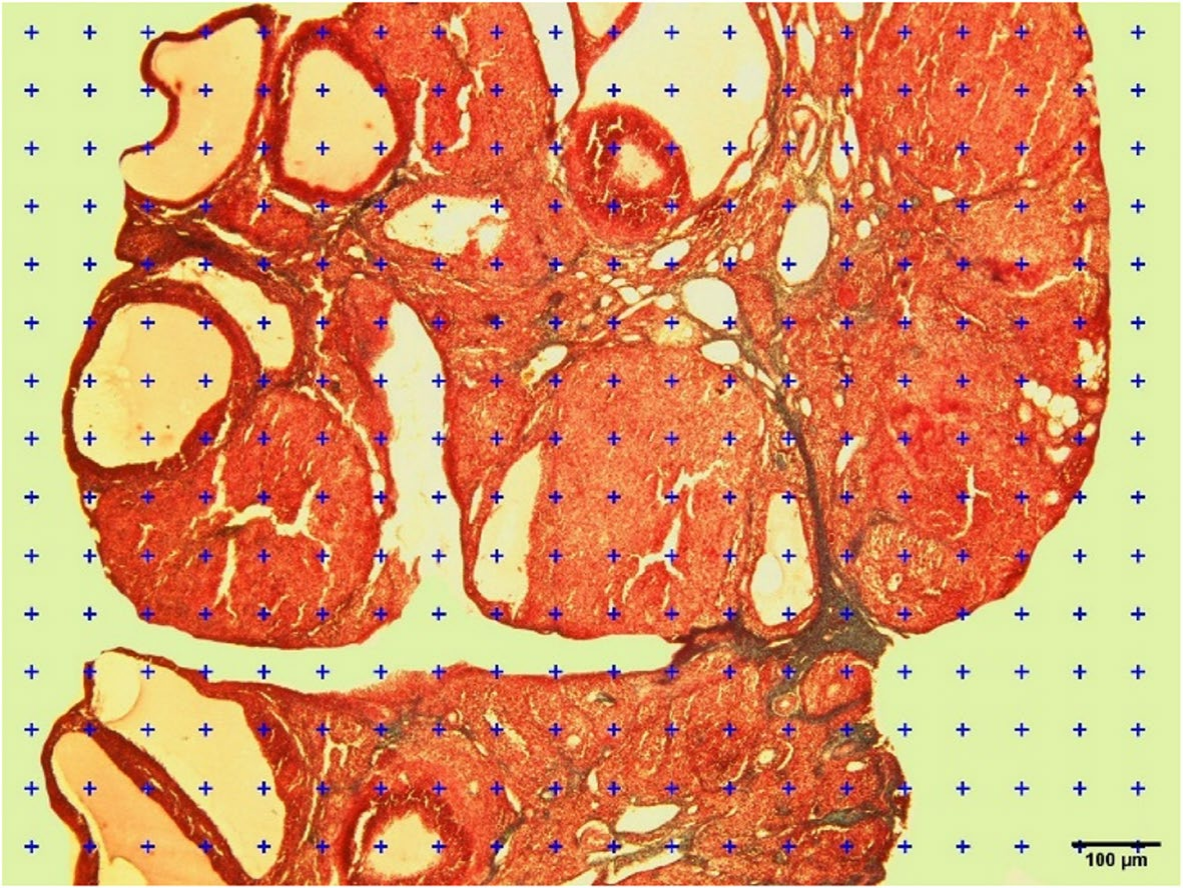
Estimation of the total volume of the ovary using the Cavalieri method. scale bar:100 um

After Masson’s Trichrome staining, collagen volume, blood vessel volume and area were calculated with the Cavalieri’s principle (Figs. 9 and 10). After quantification, the area of blood vessels in the PCOS + Stem cells group (11.26 vs. PCOS-Sham: *P* ≤ 0.02; vs. PCOS: *P* ≤ 0.001) was significantly reduced compared to the PCOS (32.74) and PCOS-Sham (24.85) groups (Fig. 12).

Moreover, the Area of blood vessels in the PCOS (Vs Control and Sham: *P* ≤ 0.0001) and PCOS-Sham (Vs Control: *P* ≤ 0.003; Vs Sham ≤ 0.004) groups significantly increased compared to the control (6.68) and Sham (6.90) groups (Fig. 12).

**Fig. 11:**
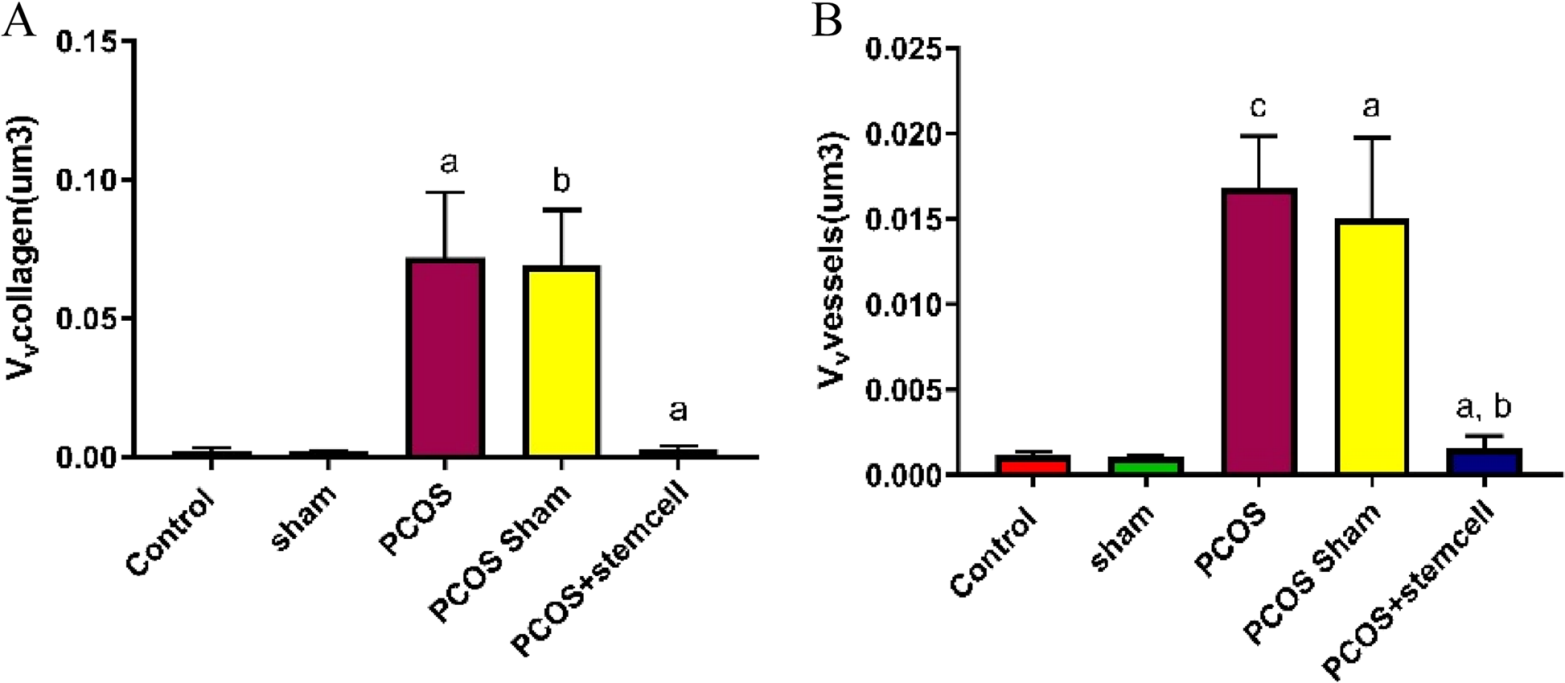
The volume of collagen (A) and blood vessels (B) in the experimental groups (V_v_vessels. **a** vs Control + Sham and PCOS-Sham; **b** vs PCOS; **c** vs Control + Sham. V_v_collagen. **a** vs Control + Sham + PCOS; **b** vs Control + sham)

**Fig. 12:**
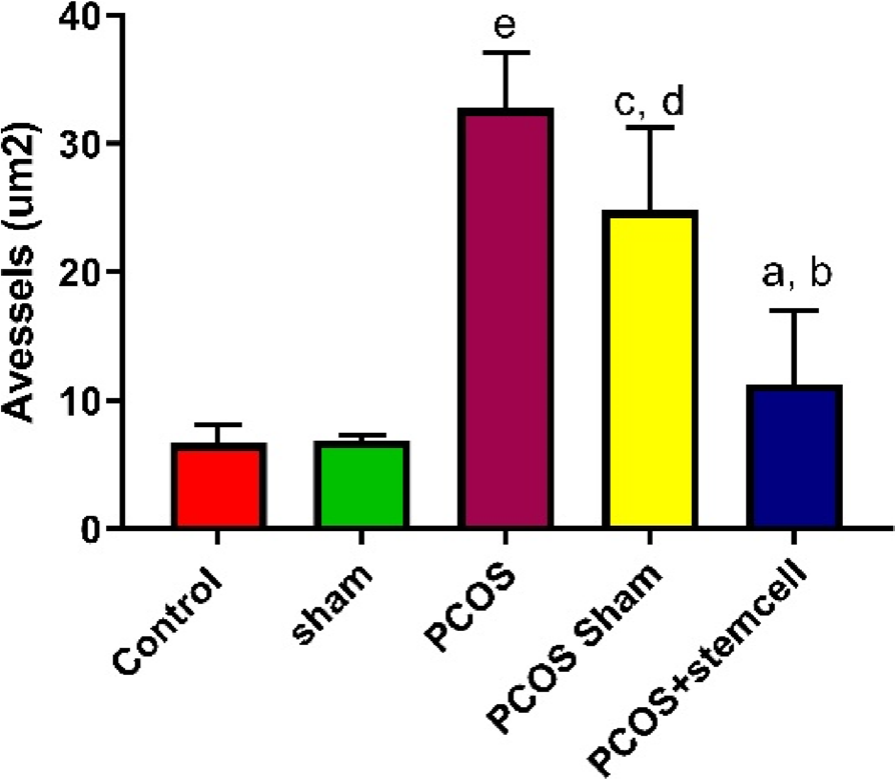
Area of blood vessels (**a** vs PCOS-Sham + PCOS; **b** vs PCOS; **c** vs Control; **d** vs Sham; **e** vs Control + Sham)

The blood vessel volume in the PCOS (0.0168, *P* ≤ 0.02) and PCOS-Sham (0.015, *P* ≤ 0.01) groups significantly increased compared to the control (0.0011) and Sham groups (0.0010) (Fig. 11A). On the contrary, in the PCOS + Stem cells group (0.0015) it appeared to be significantly reduced compared to the PCOS (*P* ≤ 0.03) and PCOS-Sham (*P* ≤ 0.01) group (Fig. 12).

The collagen volume in the PCOS (0.072, *P* ≤ 0.02) and PCOS-Sham (0.06895, *P* ≤ 0.01) groups significantly increased compared to Control (0.0011 vs. PCOS: *P* = 0.0008; vs. PCOS-Sham: *P* = 0.0011) and Sham groups (0.0010 vs. PCOS: *P* = 0.0008; vs. PCOS-Sham: *P* = 0.0011) (Fig. 11B). Differently, in the PCOS + Stem cells group (0.0029) it appeared to be significantly reduced compared to the PCOS (*P* = 0.0008) and PCOS-Sham (*P* = 0.0012) group (Fig. 11B).

### Body and ovarian weight assessment

**Fig. 13:**
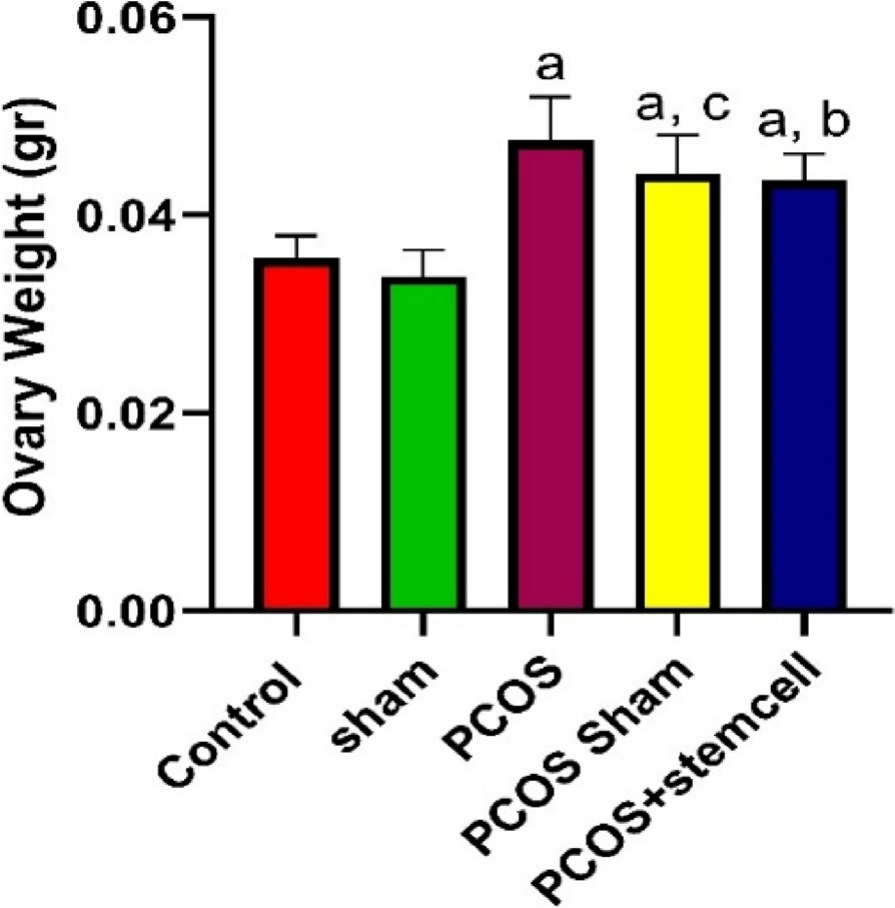
Ovarian weight in the experimental groups (**a** vs Control + Sham; **b** vs Control; **c** vs Control)

**Fig. 14:**
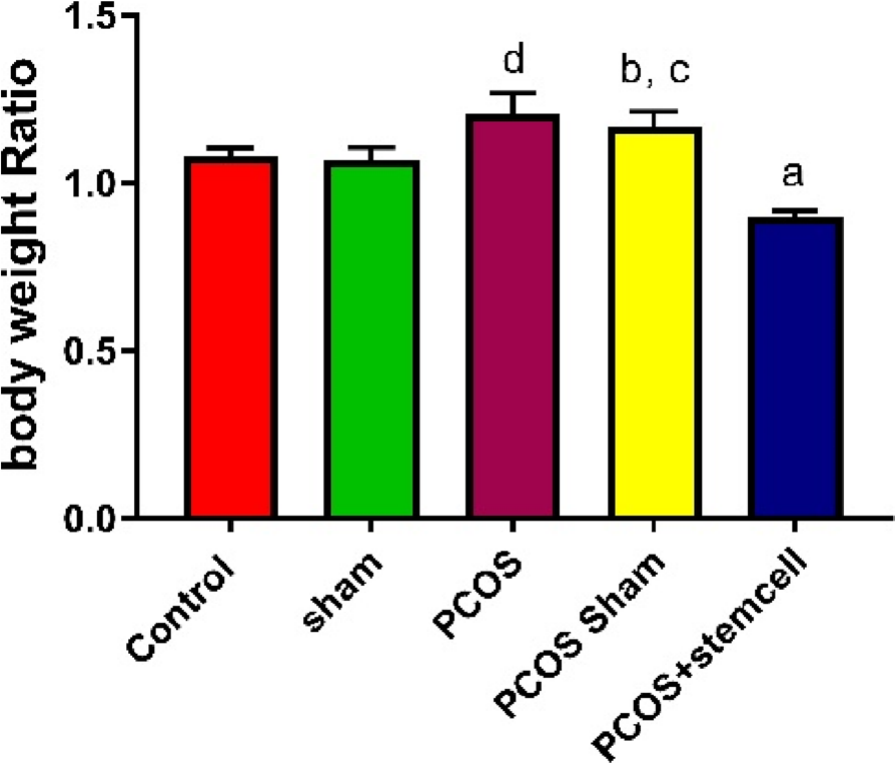
Body weight ratio in the experimental groups (**a** vs PCOS + PCOS-Sham + Sham + Control; **b** vs Sham; **c** vs Control; **d** vs Control + Sham)

The ovarian weight in PCOS (0.047) and PCOS-Sham (0.044) groups increased significantly compared to Control (0.035, vs. PCOS: *P* ≤ 0.0001; vs. PCOS Sham: *P* ≤ 0.001) and Sham (0.033, vs. PCOS and PCOS Sham: *P* ≤ 0.0001) groups. Ovarian weight reduction in the PCOS + Stem Cells group (0.043) was not significant compared to PCOS and PCOS Sham groups (Figs. 13 and 14).

Ovarian weight ratio in PCOS (1.20 vs. Control and Sham: *P* ≤ 0.0001) and PCOS-Sham (1.16 vs. Control: *P* ≤ 0.006; vs. Sham: *P* ≤ 0.002) groups significantly increased compared to Control (1.07) and Sham (1.06) groups. The observed reduction in the PCOS + Stem Cells group (0.89, vs. PCOS and PCOS Sham: *P* ≤ 0.0001) was significant compared to PCOS and PCOS Sham groups.

## Discussion

Infertility is one of the prevalent problems among couples, affecting millions of people worldwide [34]. One of the main causes of infertility in women is the Polycystic Ovary Syndrome (PCOS) [2, 35]. Despite improvements in treatment protocols, long-term drugs such as Metformin may lead to gastrointestinal problems, diarrhea and other adverse side effects that negatively affect the quality of patients’ life [18, 36].

The present study aimed to investigate the effect of human menstrual blood-derived stem cells on the ovarian histology of a PCOS model of Wistar rats. Based on the results, stem cells derived from human menstrual blood could ameliorate PCOS symptoms by improving folliculogenesis, reducing ovarian and body weight, collagen distribution and vascular volume.

The histology of vaginal smear is a determinative factor in ovarian physiology [6]. The estrus cycle of rats has four phases, including proestrus, estrus, metestrus, and diestrus. In the proestrus phase, horn cells, nucleated epithelial cells, and leukocytes can be observed. In the estrus stage, only horn cells are detected. In addition, nucleated epithelial cells and leukocytes are observed in the metestrus stage. Only leukocytes were visible in the diestrus stage. In addition, Sun and his colleagues showed that changes in the estrous cycle of rats could be due to changes in the levels of sex hormones and gonadotropins, which play an important role in regulating the estrous cycle [37].

Rats treated with letrozole were completely acyclic. The vaginal smear from this model showed a large number of leukocytes, the predominant cell type of the diestrus phase [38]. Another study showed that PCOS rats remained in diestrus for a long time while treatment with *Fagonia indica* abolished the irregularity of the estrous cycle and controlled the cycle [39].

In the present study, the results of the vaginal smear in rats demonstrated a regular estrous cycle in the control and sham groups, including the four phases of proestrus, estrus, metestrus and diestrus. Differently, in the PCOS and PCOS-Sham groups it was found an irregularity in the estrous cycle with a longer diestrus. The noted finding is in line with the study of Caligioni and coworkers in the same experimental model [40].

In PCOS patients, small antral follicles are unable to reach the ovulatory phase, thus turning into ovarian cysts. Considering that receptors of various hormonal factors are expressed on the oocyte and follicular cells with the different growth and development of follicles, any change or occupation of these receptors can lead to abnormal functioning of follicles and developmental disorders. Reduced or abnormal levels of hormones as FSH and LH, defects in specific local factors and abnormal steroidogenesis in the ovaries are the main causes of follicular growth arrest, as occurs in PCOS [41]. Therefore, follicular cells gradually enlarge, without further mitosis, to then switch to luteal granulosa cells. PCOS patients suffer from chronic anovulation, and this causes a decrease in the number of corpora lutea, as found in PCOS animals [25].

One of the main features of polycystic ovary is significant increasing in the number of follicles that related with the higher population of developing preantral and antral follicles than that of normal ovaries. The process of excessive formation of follicles in PCOS ovaries has not been confirmed; nonetheless, several possibilities have been proposed, including increased activation of primordial follicles, slowing of preantral follicle growth, increased follicle survival, and/or reduced atresia or a combination of these processes [38].

Hughesdon and colleagues indicated a 2-fold increase in the number of developing follicles [42], as one of the first descriptions of PCOS ovaries. These findings indicated that the increased number of preantral and primary antral follicles in polycystic ovaries could not be due to an increased primary reservoir or an accelerated rate of primary follicle activation. Further studies found a raising in the number of total follicles, but determining of the size of the initial pool has been inconsistent [43]. The growth of preantral follicles occurs more slowly in PCOS, leading to an accumulation of growing follicles that can explain this phenomenon. This possibility is consistent with the study of the group of Maciel in which the number of primordial follicles was similar in PCOS and normal ovaries, while that of growing preantral follicles was greater in PCOS [44].

In the present study, we applied Letrozole to induce a PCOS model in rats where the follicular growth was inhibited, atresia increased, concomitantly with the presence of large cysts without a granulosa cell layer or large cystic follicles with few granulosa cells, in agreement to others [6, 25]. The advantage of our study compared to other studies is the type of cell source used to treat PCOS. It has been reported that PCOS can be associated with insulin resistance and obesity. However, the exact cause is unknown. Most PCOS women experience insulin resistance, with a 50–90% incidence, thus suggesting that insulin resistance is an important mediator among PCOS and dysmetabolic diseases. Insulin resistance and hyperinsulinemia can increase androgen secretion and reduce the level of sex hormone-binding globulin (SHBG), thus leading to hyperandrogenemia, weight increase and obesity [45]. Additionally, an increase in androgen levels leads to a hypertrophic condition in fat cells, due to the expression of enzymes and proteins involved in carbohydrate and lipid metabolism, and oxidative stress [46, 47]. Compared to healthy women, PCOS patients have higher obesity, longitudinal weight, and central obesity [48, 49]. Based on our findings, the weight of PCOS animals increased compared to controls, in agreement with others [11]. In addition, Karimzadeh and colleagues showed an increase in body and ovarian weight in rats with PCOS [50].

MSCs can modulate innate immune responses by interacting with various immune cells, including inhibiting the proliferation of T cells, B cells, dendritic cells (DCs), natural killers (NKs), and regulatory T cells (Tregs) [51, 52]. Currently, many advances occurred in elucidating the immunological properties of BM-MSCs. However, studies on the immune system of MenSCs are relatively rare compared with BM-MSCs. There are some similarities between BM-MSCs and MenSCs. Nevertheless, there are still differences in the extrapolation of functional or regenerative properties. Based on another study, MenSCs have stronger immunomodulatory properties compared to UC-MSCs as indicated by the inhibition of T cell proliferation [53]. Following further evaluation, MenSCs increased the survival of xenograft-versus-host disease (GVHD) in mice/rats by limiting the proliferation of CD4 + IFN-γ + or CD8 + IFN-γ + T cells that exert an immunosuppressive function [54]. Bozorgmehr et al. demonstrated that MenSCs generate an immune-modulating effect by blocking the production and maturation of dendritic cells and the secretion of interleukin (IL)-6 and IL-10 [55].

Recently, several studies focused on the regulatory effects of fibrotic factors such as MMPs and TIMPs on the ECM balance in PCOS patients, because there is an opinion that these factors play an important role in the growth disorder and follicular atresia of PCOS by helping the production of ovarian stromal components. Moreover, the abnormal expression of TGF-β1 may be related to the ovary pathogenesis of PCOS and the increment of TGF-β1 level in the follicular fluid [56]. In the ovarian tissue, theca cells, responsible for the secretion of androgens in normal ovaries, play a pivotal role in maintaining the integrity of the follicle and its function. Abnormal expression of TGF-β1 causes interstitial cell overgrowth, leading to increased androgen production in PCOS patients. Furthermore, the overexpression of TGF-β1 disrupts the balance between MMPs and TIMPs which causes excessive accumulation of ECM [57].

It has been reported that an increase in ovarian mass is associated with an abnormal increase in blood vessels in PCOS. This condition is attributed to an abnormal expression of several angiogenesis factors, such as VEGF, angiopoietin-1 and − 2, platelet-derived growth factor (PDGF), transforming growth factor beta (TGF-β), and basic fibroblast growth factor (bFGF). A 2004 study demonstrated the abnormal expression of TGF-β1 in the ovary and its role in the pathogenesis of PCOS [58]. We here found an increase in the volume and area of blood vessels in the PCOS and PCOS Sham groups, in agreement with the study of Zaidi and coworkers [59] In our study, increased collagen volume was observed in the PCOS and PCOS Sham groups, thus reinforcing the possible role of TGF-β1 into ovarian tissue capsule fibrosis in PCOS [60]. Subsequently, TGF-β1 may lead to excessive accumulation of ECM in the ovary and the development of ovarian interstitial fibrosis [61].

## Conclusion

This study showed that human menstrual blood-derived stem cellsimprove the function of folliculogenesis in rats with polycystic ovaries. This could be due to the secretion of paracrine factors from these cells, which can improve PCOS symptoms by improving fibrosis as well as angiogenesis and obesity.

## Data Availability

The data used to support the finding of current study are available from the corresponding author, on reasonable request.
